# Butterfly declines in protected areas of Illinois: Assessing the influence of two decades of climate and landscape change

**DOI:** 10.1371/journal.pone.0257889

**Published:** 2021-10-13

**Authors:** Nicole B. Kucherov, Emily S. Minor, Philip P. Johnson, Doug Taron, Kevin C. Matteson

**Affiliations:** 1 Department of Biology/Project *Dragonfly*, Miami University, Oxford, OH, United States of America; 2 Biological Sciences (M/C 066), University of Illinois at Chicago, Chicago, IL, United States of America; 3 Chicago Academy of Sciences/Peggy Notebaert Nature Museum, Chicago, IL, United States of America; Southeastern Louisiana University, UNITED STATES

## Abstract

Despite increasing concern regarding broad-scale declines in insects, there are few published long-term, systematic butterfly surveys in North America, and fewer still that have incorporated the influence of changing climate and landscape variables. In this study, we analyzed 20 years of citizen science data at seven consistently monitored protected areas in Illinois, U.S.A. We used mixed models and PERMANOVA to evaluate trends in butterfly abundance, richness, and composition while also evaluating the effects of temperature and land use. Overall butterfly richness, but not abundance, increased in warmer years. Surprisingly, richness also was positively related to percent impervious surface (at the 2 km radius scale), highlighting the conservation value of protected areas in urban landscapes (or alternately, the potential negative aspects of agriculture). Precipitation had a significant and variable influence through time on overall butterfly abundance and abundance of resident species, larval host plant specialists, and univoltine species. Importantly, models incorporating the influence of changing temperature, precipitation, and impervious surface indicated a significant overall decline in both butterfly abundance and species richness, with an estimated abundance decrease of 3.8%/year and richness decrease of 1.6%/year (52.5% and 27.1% cumulatively from 1999 to 2018). Abundance and richness declines were also noted across all investigated functional groups except non-resident (migratory) species. Butterfly community composition changed through time, but we did not find evidence of systematic biotic homogenization, perhaps because declines were occurring in nearly all functional groups. Finally, at the site-level, declines in either richness or abundance occurred at five of seven locations, with only the two largest locations (>300 Ha) not exhibiting declines. Our results mirror those of other long-term butterfly studies predominantly in Europe and North America that have found associations of butterflies with climate variables and general declines in butterfly richness and abundance.

## Introduction

There is increasing concern regarding potential broad-scale declines in insects [1–3]. Substantial declines have been reported for biomass of flying insects in Germany [4], butterflies in Europe [5], and for abundance of macro moths, beetles, and caddisflies in the Netherlands [6]. Although these and other similar studies have been widely cited and reported on in the media, it is clear that insect declines are not uniformly occurring across all taxa, regions, or time periods [7, and references therein]. For example, a global study including over 1600 sites across 41 countries found a decline in terrestrial insects, but an increase in freshwater insect abundance [8]. A synthesis by Pilotto et al. [9] also found a decline of terrestrial insects but an increase in freshwater insect diversity (not abundance). Furthermore, a recent U.S. study found declines of some insect taxa but no net declines across all groups [10]. Clearly, additional studies are needed to understand nuanced regional insect trends, as well as to disentangle potential causal factors including habitat conversion and climate change.

Butterflies are an excellent group to monitor and assess due to their relative ease of identification, status as ‘charismatic microfauna’, and the availability of data generated by citizen science efforts (e.g., UK Butterfly Monitoring Scheme, North American Butterfly Association, PollardBase programs). European studies have generally indicated that butterflies are declining. In Britain, 34 of 46 butterfly species declined over a 30-year period (1970–1999) [11], while a 10% decline in butterfly species richness occurred in Denmark over a century (1900–2012) [12]. A 39% decrease was documented in Germany from 1840 to 2013 [13] and a similarly severe decline was noted in the Netherlands from 1890 to 2017 [14]. An additional study, conducted in Spain, found that 70% of butterfly species declined from 1994 to 2014 [15].

In North America, we are aware of only a few long-term (multi-decadal) analyses of entire butterfly communities: a 45-year dataset initiated by Arthur Shapiro in California [16, 17], an analysis of citizen butterfly surveys from 1992–2010 in Massachusetts [18], a 20-year dataset derived from citizen science efforts in Ohio [19], and most recently, a large-scale analysis of citizen science data for nine ecoregions across North America [20]. The California work demonstrated species richness reductions at 5 of 10 sites over 35 years, with declines being most notable at lower-elevation locations with greater habitat loss [16] and frequent use of neonicotinoids [17]. More recently, this dataset was combined with citizen science data from the North American Butterfly Association and iNaturalist and indicated a 1.6% annual decline in butterfly abundance over four decades in the western United States [21]. The work in Massachusetts indicated declines in northern butterflies [18] while the study in Ohio found a 33% cumulative decline in butterfly abundance from 1996 to 2016 [19]. Although the Ohio study did not directly evaluate the influence of climate or habitat change, the authors suggested that potential drivers of the decline could include degradation of remaining butterfly habitat, climate-related shifts from cool-adapted to warm-adapted species, and increased use of pesticides and herbicides [19]. The nation-wide citizen science analysis indicated substantial heterogeneity in butterfly abundance across ecoregions of the United States [20], with some regions experiencing increases in butterfly abundance while others declined.

Landscape-scale studies have suggested potential drivers of butterfly decline and community change. For example, local butterfly species richness increased with the percentage of surrounding semi-natural grassland (3 km radius scale) in Sweden [22] and surrounding butterfly habitat (250 m radius scale) in Ontario, Canada [23]. In contrast, negligible effect of surrounding landscape (building units at the 500 m radius scale) on butterfly richness was found in New York City [24]. In Illinois (USA), monarch (*Danaus plexippus*) abundance was negatively correlated with county-level applications of the herbicide glyphosate [25]. Climate variables, too, appear to have discordant effects on butterfly communities. While butterflies typically respond positively to warmer temperatures [26], they can exhibit variable responses to precipitation [27, 28]. These and other studies indicate the importance of including landscape context (e.g., impervious surface and crop lands) and climate variables when assessing trends in butterfly abundance and richness.

In addition to changes in abundance and richness over time, butterfly communities have been shown to shift in composition from habitat or host plant specialists to more generalists [12], which could result in biotic homogenization [29, 30]. Previous research has found biotic homogenization of butterfly populations increases with high agricultural intensity [31, 32] and increasing urbanization [33]. One study that examined butterfly records collected over more than a century found pervasive homogenization caused by severe declines in specialist species [12]. Alternately, homogenization may be less apparent if both generalists and specialists decline, as has been found for butterfly communities in a variety of contexts [15, 19, 34]. Degree of residency may also be a factor in butterfly population trends or lack thereof. In Ohio, the monarch was the only species to exhibit a statistically significant decline out of 14 migratory species [19]. Migratory butterfly populations can be irruptive in some years [16, 19], with high inter-annual variation making it less likely to see significant trends through time.

In this study, we assess data collected by the Illinois Butterfly Monitoring Network (IBMN) to determine trends in butterfly communities in Illinois, USA, over a 20-year period (1999–2018). We examined potential drivers of community change, including precipitation, temperature, and land cover change. We also investigated shifts in overall butterfly community composition, the degree of biotic homogenization through time, and changes in butterfly functional group abundance and richness. Based on the literature, we hypothesized that there would be a decrease in butterfly abundance and species richness over the last two decades in Illinois. Furthermore, we hypothesized that declines would be greater for specialists than generalists, resulting in biotic homogenization.

## Methods

### Study sites and data collection

Data were collected through the Illinois Butterfly Monitoring Network (IBMN; https://bfly.org/), which has been monitoring butterflies in Illinois since 1987. The network consists of a coordinator and a group of volunteers. The protocol is to use Pollard transect walks to record all species sighted within a 6-m radius of transects between the hours of 10 AM and 3:30 PM on days with less than 50% cloud cover [35]. All citizen-collected data are deposited in PollardBase (https://www.pollardbase.org/), an online repository of Pollard survey data. Following submission, data are reviewed by an IBMN administrator. Species reports that seem unlikely for a given location or time are either confirmed (via photo or follow-up communications with observers) or removed from the dataset.

**Fig 1:**
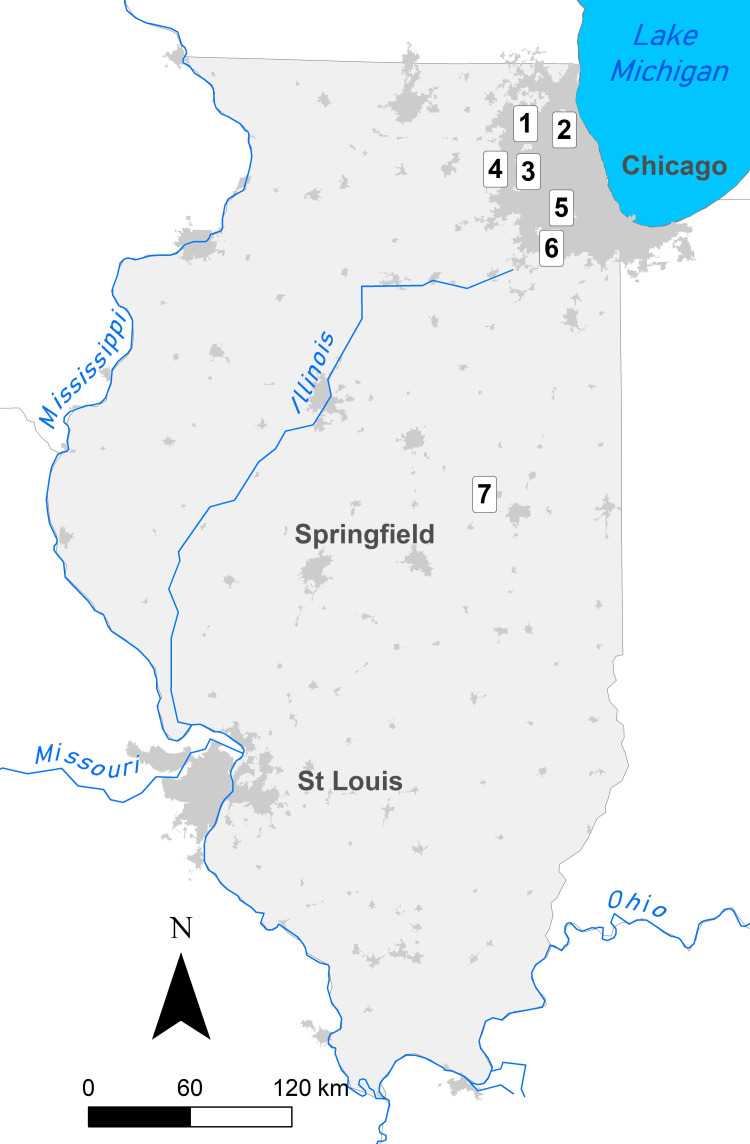
Map of study sites in Illinois. State of Illinois (light gray polygon) including locations of the seven study sites: (1) Cuba Marsh, (2) Somme Prairie Grove, (3) Bluff Spring Fen, (4) Ferson Creek Fen, (5) Spears Woods, (6) Hickory Creek Barrens, (7) Buffalo Trace. Dark grey areas indicate the locations of cities throughout the state, with major cities labeled. Map data layers meet requirements for Creative Commons Attribution License (CCAL) CC BY 4.0 and come from the following sources: (1) rivers: National Weather Service, Rivers of the US (subset); (2) state boundaries and urban areas: cartographic boundary files from the US Census Bureau’s MAF/TIGER geographic database.

Because our goal was to assess trends in specific functional groups and homogenization over time, we restricted our analyses to the seven most consistently monitored sites in Illinois, USA (Fig 1). These sites are all public conservation lands. For inclusion in our study, sites were required to have at least three suitable study years in each of the following five-year time periods: 1999–2003, 2004–2008, 2009–2013, and 2014–2018. Since few observations occurred during May (in the earlier years) and August (in later years), we restricted our study to sites with at least three observation dates between June 1 and July 31. Two sites each had a single study year with only three observation dates (Somme Prairie Grove in 2011 and Ferson Creek Fen in 2015); all other study years, and all other study sites, had at least four observation dates per year between June 1 and July 31. If sites had more than four observation dates per year within this time period, we retained the first and last sampling dates, and selected two other dates that maximized temporal spread between observations. In this way, we standardized the sampling effort as much as possible between sites and years without any interpolation or extrapolation of data. Based on these criteria, we included six locations in northeastern Illinois as well as one site in central Illinois (Fig 1; Table 1). The landscape surrounding these sites is predominantly suburban (the six sites in Cook, Lake, Kane and Will counties) or agricultural (the site in Champaign County). The seven locations were an average of 86 km apart, with a median separation distance of 54 km (Range = 11 to 220 km).

**Table 1:** Characteristics of the seven butterfly monitoring locations in Illinois, USA.

Site	County	Size (Ha)	Transect length (km)	Average sampling days per year	Percentage of years sampled (1999–2018)
Cuba Marsh (Lake County Forest Preserve)	Lake	316	3.80	4.00	95%
Somme Prairie Grove	Cook	34	1.25	3.94	85%
Bluff Spring Fen	Cook	40	2.27	4.00	95%
Ferson Creek Fen	Kane	17	1.95	3.94	80%
Spears Woods	Cook	190	2.03	4.00	100%
Hickory Creek Barrens	Will	624	2.51	4.00	90%
Buffalo Trace Prairie (Lake of the Woods Forest Preserve)	Champagne	108	3.54	4.00	75%

Overall, the transect routes and sampling effort for the included sites remained unchanged over the 20-year period (1999–2018; Table 1), providing an ideal dataset for assessing the influence of climate and landscape variables, without confounding effects of differences in sampling effort or changes in locations. Over the 20-year period at these seven locations, citizen volunteers walked 1223 km, monitored butterflies for 51,000 minutes, and recorded 31,622 individual butterflies, providing a substantial dataset for these analyses.

### Butterfly richness, abundance, and functional groups

All butterflies were identified to species with the exception of *Celastrina ladon/neglecta* and *Colias eurytheme/philodice*, each of which were treated as a single taxon due to species-level identifications being unreliable (S1 Appendix). For abundance measures, we included all observed individuals regardless of species determination. For each site, we calculated the annual species richness (cumulative, over all sample dates in a year) and abundance (average of all sampling days). Using Lotts & Naberhaus [36] (and references therein), we classified butterfly species by residency status for Illinois (resident or non-resident), and larval host plant specialization (generalist or specialist, with specialists using host plants from only one family or genus). We confirmed these classifications for the Illinois region, and determined the number of generations per year (univoltine or multivoltine, with multivoltine group including bivoltine species) using Jeffords et al. 2019 [37] for butterflies and Bouseman et al. 2006 [38] for skippers (S1 Appendix).

### Land use change and climate data

The percentage of impervious surface and crop cover within a 2 km radius around each site were each determined using the National Land Cover Database (NLCD) [39] for the years available within our study period; 2001, 2006, 2011, and 2016. Land cover variables were relatively consistent through time for most sites (S3 Appendix). Therefore, for years in which no data was available from the NLCD, we used the closest year available (e.g., the 2001 value was used for years between 1999 and 2003). Climate data were derived from Daymet (https://daymet.ornl.gov/) in 1 km gridded cells, using the endpoint of each sampling transect to select the cell. We extracted daily precipitation, daily maximum temperature and daily minimum temperature, then calculated the yearly average from October 1 to September 30 (i.e., the ‘water year’) for each site and year over the 20-year sampling period. While we explored the possibility of quantifying management activities at the sites, it was not possible to do so due to staff changes and variable record-keeping at the sites, as well as differences in the spatial extent, timing, and frequency of burns, invasive plant removal, native plantings, etc.

### Statistical methods

#### Trends in abundance, richness, and functional groups

We used a model selection approach, with generalized linear mixed effects models (GLMM), to examine the effect of time, climate, and land cover on total butterfly richness, abundance, and functional groups. Response variables included annual species richness and abundance per site. Additionally, we examined the species richness and abundance for each of our functional groupings: resident/non-resident species, host plant specialist/generalist species, and univoltine/multivoltine species.

Potential predictor variables included time (i.e. years since 1999) and the land cover and climate variables described previously. Prior to running the models, we used repeated measures correlation (R package rmcorr) [40] to evaluate relationships among all variables. Two variable pairs had correlation coefficients above 0.80: average temperature maximum and average temperature minimum (r = 0.923), and impervious surface and crop cover (r = 0.831) (S4 Appendix). To avoid multicollinearity in our models, we excluded crop cover and average temperature minimum as independent variables but interpret our results with these correlations in mind. We standardized all remaining continuous variables through mean centering and scaling by standard deviations to aid in model convergence. Due to the correlation between max and minimum temperature, we refer to the average temperature maximum variable as ‘temperature’ hereafter. For simplicity, ‘precipitation’ refers to the average daily precipitation across the water year for each site and year.

For each response variable individually, we started with a global model that included all possible predictor variables: two climate variables (temperature and precipitation), a land cover variable (percent impervious surface), and time (years since 1999), as well as interactions between time and each of the other variables. “Site” and “year” were included as random effects to account for repeated measures and slight differences in the number of sites sampled per year. For each model, we used a stepwise deletion procedure to identify the best model. Variables were dropped from the global model if they were not significant (p > 0.05) and if their removal increased support for the model based on a lower AIC_c_.

We initially fit models with a log-linked Poisson distribution. However, if model residuals were over or under dispersed at an alpha of 0.05 with the Poisson distribution, a negative binomial distribution was used. We visually inspected all model residuals. Analyses were run in R 4.0 using the packages lme4 [41] and lmerTest [42].

#### Community composition & biotic homogenization

We evaluated the effects of climate, surrounding impervious surface, and time on butterfly community composition using a permutational multivariate analysis of variance (PERMANOVA) with Bray-Curtis dissimilarity and 999 permutation constrained within survey sites. The PERMANOVA was conducted using the *adonis* function in the R package *vegan* [43]. Although these sites were consistently monitored, there were missing years for some sites which could affect composition and similarity indices. Therefore, to test for both composition change and biotic homogenization (see below) of butterfly communities from 1999 to 2018, we evaluated beta diversity between communities over four 5-year periods (1999–2003, 2004–2008, 2009–2013, 2014–2018). Beta diversity was estimated using multivariate dispersion of species composition in each time period, which measures the dispersion of each community from the centroid of all communities considered in a period [44]. If butterfly communities are homogenizing, we would expect less dispersion in more recent time periods. Dispersion of each community from the period centroid was calculated using the *betadisper* function in the R package *vegan* [43]. Differences in community dispersion were evaluated using a permutation-based test of multivariate homogeneity of group variances with *permutest* in *vegan* [43] using 999 permutations.

## Results

A total of 74 butterfly species were observed at the seven sites over the study period (S1 Appendix). Nineteen species made up 90% of the total abundance. The five most abundant taxa across all sites were the Cabbage White *(Pieris rapae)* (23% of observations), Common Wood Nymph (*Cercyonis pegala)* (9%), Pearl Crescent (*Phyciodes tharos*) (9%), Great Spangled Fritillary *(Speyeria cybele)* (7%), and Spring/SummerAzure *(Celastrina ladon/neglecta)* (7%) (S1 Appendix). The majority of butterfly species were residents (77%), host plant specialists (76%), and multivoltine (64%).

On average, 23% of the landscape in a 2 km radius surrounding the seven sites was impervious surface (Range = 14.3 to 34.1%; S3 Appendix). Although impervious surface increased through time (S4 Appendix), the amount remained relatively stable at most individual sites (S3 Appendix), with two sites experiencing larger shifts: Bluff Spring Fen increased from 25.8% to 34.1%, while Hickory Creek Barrens increased from 17.3% to 26.7%. On average, 7% of the landscape surrounding the seven sites was crop cover (Range = 0.1 to 25.9%; S3 Appendix). Crop cover significantly decreased through time (S4 Appendix) with Hickory Creek Barrens seeing the largest drop (17.1% to 5.8%; S3 Appendix).

Across all seven sites, the average daily precipitation over the 20 years was 2.8 mm/day (range = 1.9 to 4.3 mm/day), the average daily maximum temperature was 15.5°C (range = 12.6 to 19.5) and the average daily minimum temperature was 4.8°C (range = 1.7 to 7.2) (S4 Appendix). Average daily precipitation significantly increased across all years from 1999–2018 (S4 Appendix). There was not a significant change over time for average daily maximum temperature (S4 Appendix).

### Changes in overall species richness and abundance

**Fig 2:**
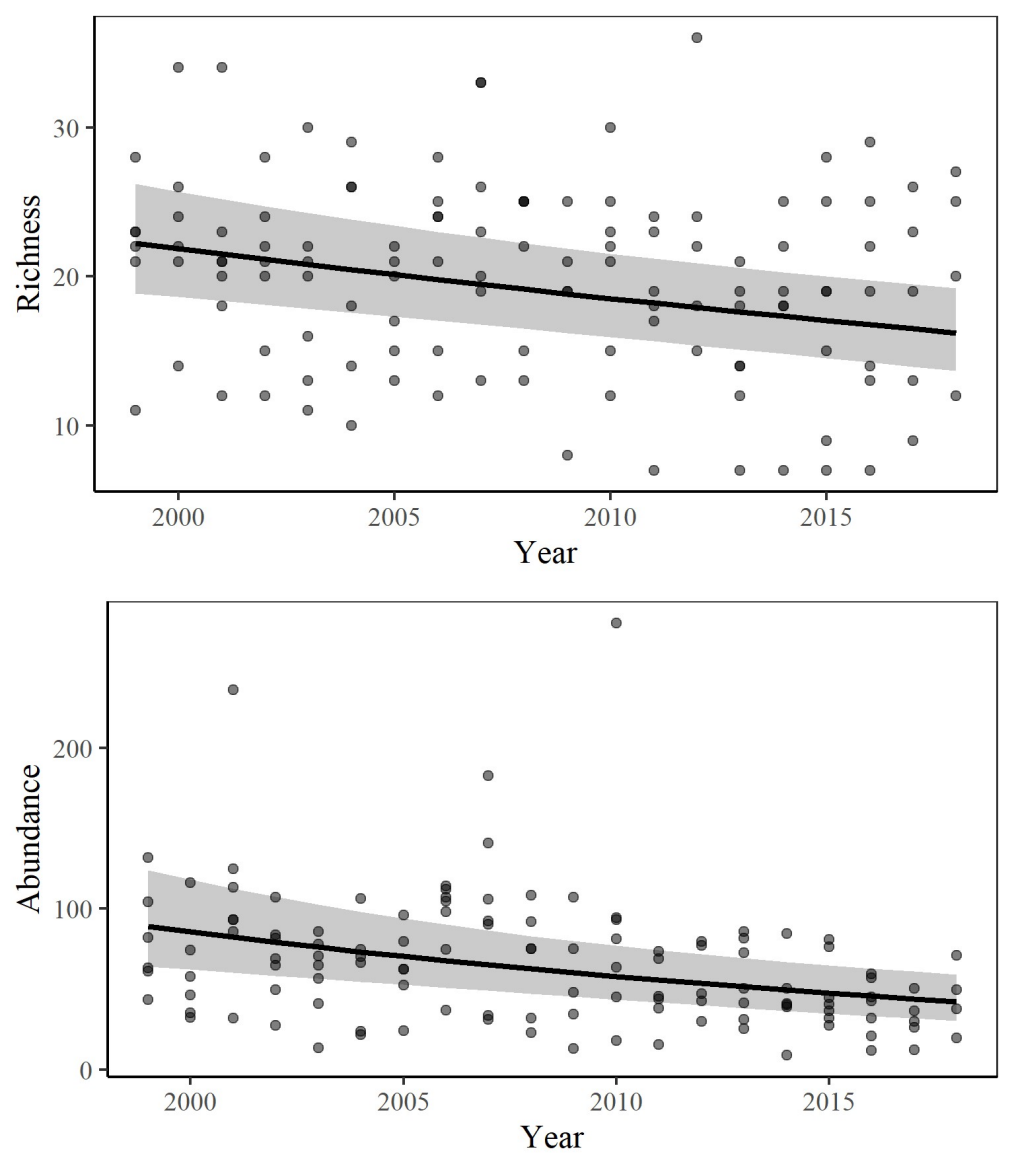
Model estimates for overall abundance and richness. Estimates are across all seven sites over time, using the overall mean for all other fixed effects. The black squares represent the point estimates, error bars show 95% confidence intervals, and open circles dots are the observed means.

The final models for overall butterfly abundance and richness indicated that both measures significantly declined through time (Table 2; Fig 2). For butterfly abundance, there was a significant negative interaction of time and precipitation. Butterfly species richness was positively associated with temperature and surrounding impervious surface (Table 2).

**Table 2:** Model selection results for variables influencing butterfly abundance and richness at seven sites in Illinois from 1999–2018.

	Estimate	S.E.	Z-value	P value
**Abundance** (*Rm*^*2*^ *=* 0.17; *Rc*^*2*^ *=* 0.57; AICc = 1169.06; ΔAICc = 7.63)				
Intercept	4.12	0.14	28.49	<0.01
Time	-0.23	0.05	-4.19	<0.01
Precipitation	-0.01	0.05	-0.15	0.88
Time: Precipitation	-0.12	0.06	-2.18	0.03
**Richness** (*Rm*^*2*^ *=* 0.22; *Rc*^*2*^ *=* 0.55; AICc = 708.63; ΔAICc = 8.09)				
Intercept	2.94	0.08	38.66	<0.01
Time	-0.10	0.02	-4.11	<0.01
Temperature	0.06	0.03	2.25	0.02
Impervious surface	0.15	0.06	2.58	<0.01

Generalized linear mixed effects models were used, with site and year included as a random effect. The most parsimonious and explanatory model, as identified by the lowest AIC_c_ value, is shown. Estimates are standardized. Marginal R^2^ (*Rm2)* and conditional R^2^ (*Rc*^*2*^*)* and are shown for each model as well as AIC_c_ and ΔAIC_c_ (difference in AIC_c_ from the global model). ‘Precipitation’ is the average daily precipitation per site from October 1 to September 30 and ‘Temperature’ is the average daily maximum temperature for this same time period. ‘Impervious surface’ was measure in a 2 km radius around each site and year.

From 1999 to 2018, there was an estimated abundance decrease of 3.8%/year and richness decrease of 1.6%/year (52.5% and 27.1% cumulatively). When examined individually, four of the sites experienced significant declines in butterfly abundance and two sites experienced significant declines in species richness (S2 Appendix). None of the locations significantly increased in butterfly abundance or richness through time.

### Changes in functional groups

Declines in butterfly abundance and/or richness were observed in all functional groups except non-resident species (Table 3). Impervious surface had a positive influence on richness of all butterfly functional groups except larval host plant generalists. Temperature had a positive effect on richness of non-residents, larval host plant specialists, and bi/multivoltine species. Conversely, temperature had a negative effect on the abundance of univoltine species. Precipitation by itself did not enter into many of the final models except for a marginal negative relationship with non-resident abundance (p = 0.08). However, the interaction of time and precipitation had a significant influence on abundance of resident butterflies, larval host plant specialists, and univoltine species. These interactions generally indicate that precipitation has had an increasingly negative effect on butterfly abundance over time, with more recent years being more negatively affected by increased average precipitation than earlier years. For univoltine abundance only, there was a significant interaction of time and temperature and a significant interaction of time and impervious surface.

**Table 3:** Model selection results for drivers of butterfly functional group abundance and richness.

	Estimate	S.E.	Z value	P value
*Resident species (N = 57 species)*				
Abundance (*Rm*^*2*^ *=* 0.18; *Rc*^*2*^ *=* 0.54; AICc = 1150.65; ΔAICc = 6.93)				
Intercept	4.04	0.14	28.07	<0.01
Time	-0.23	0.05	-4.56	<0.01
Precipitation	0.00	0.05	0.04	0.97
Time: Precipitation	-0.14	0.05	-2.75	<0.01
Richness (*Rm*^*2*^ *=* 0.20; *Rc*^*2*^ *=* 0.53; AICc = 668.39; ΔAICc = 8.69)				
Intercept	2.78	0.08	34.05	<0.01
Time	-0.10	0.02	-4.02	<0.01
Impervious surface	0.15	0.06	2.46	0.01
*Non-resident species (N = 17 species)*				
Abundance (*Rm*^*2*^ *=* 0.02; *Rc*^*2*^ *=* 0.81; AICc = 580.55; ΔAICc = 8.08)				
Intercept	1.25	0.28	4.40	<0.01
Precipitation	-0.15	0.08	-1.78	0.08
Richness (*Rm*^*2*^ *=* 0.12; *Rc*^*2*^ *=* 0.18; AICc = 432.31; ΔAICc = 9.46)				
Intercept	1.14	0.07	16.80	<0.01
Temperature	0.20	0.07	2.88	<0.01
Impervious surface	0.14	0.07	2.13	0.03
*Larval Host Plant Specialist (N = 56 species)*				
Abundance (*Rm*^*2*^ *=* 0.11; *Rc*^*2*^ *=* 0.73; AICc = 1038.72; ΔAICc = 4.93)				
Intercept	3.55	0.23	15.47	<0.01
Time	-0.24	0.05	-4.71	<0.01
Precipitation	0.04	0.05	0.81	0.42
Time: Precipitation	-0.13	0.05	-2.42	0.02
Richness (*Rm*^*2*^ *=* 0.25; *Rc*^*2*^ *=* 0.46; AICc = 650.35; ΔAICc = 7.15)				
Intercept	2.57	0.07	36.76	<0.01
Time	-0.10	0.03	-3.70	<0.01
Temperature	0.07	0.03	2.52	0.01
Impervious surface	0.17	0.06	2.98	<0.01
*Larval Host Plant Generalist (N = 18 species)*				
Abundance (*Rm*^*2*^ *=* 0.06; *Rc*^*2*^ *=* 0.46; AICc = 986.42; ΔAICc = 10.46)				
Intercept	3.03	0.17	17.93	<0.01
Time	-0.19	0.08	-2.37	0.02
Richness (*Rm*^*2*^ *=* 0.02; *Rc*^*2*^ *=* 0.32; AICc = 528.90; ΔAICc = 10.93)				
Intercept	1.78	0.11	16.78	<0.01
Time	-0.06	0.04	-1.86	0.06
*Univoltine (N = 27 species)*				
Abundance (*Rm*^*2*^ *=* 0.33; *Rc*^*2*^ *=* 0.74; AICc = 986.42; ΔAICc = 0.00)				
Intercept	2.70	0.23	11.64	<0.01
Time	-0.36	0.06	-5.89	<0.01
Precipitation	-0.01	0.05	-0.12	0.90
Temperature	-0.17	0.06	-2.79	<0.01
Impervious surface	0.36	0.16	2.29	0.02
Time: Precipitation	-0.17	0.06	-2.76	<0.01
Time: Temperature	0.14	0.06	2.62	<0.01
Time: Impervious surface	-0.12	0.05	-2.31	0.02
Richness (*Rm*^*2*^ *=* 0.22; *Rc*^*2*^ *=* 0.41; AICc = 487.84; ΔAICc = 9.45)				
Intercept	1.54	0.10	14.85	<0.01
Time	-0.19	0.05	-4.24	<0.01
Impervious surface	0.24	0.09	2.63	<0.01
*Bi/Multivoltine (N = 47 species)*				
Abundance (*Rm*^*2*^ *=* 0.13; *Rc*^*2*^ *=* 0.54; AICc = 1092.59; ΔAICc = 9.88)				
Intercept	3.67	0.14	27.07	<0.01
Time	-0.15	0.07	-2.02	<0.01
Temperature	0.15	0.09	1.58	0.11
Richness (*Rm*^*2*^ *=* 0.16; *Rc*^*2*^ *=* 0.41; AICc = 662.95; ΔAICc = 8.01)				
Intercept	2.67	0.07	38.74	<0.01
Time	-0.06	0.03	-2.28	0.02
Temperature	0.07	0.03	2.69	<0.01
Impervious surface	0.12	0.06	2.18	0.03

General linear mixed effects models were used, with site and year included as a random effect. Only the most parsimonious and explanatory model, as identified by the lowest AIC_c_ value, is shown. Estimates are standardized. Marginal R^2^ (*Rm2)* and conditional R^2^ (*Rc*^*2*^*)* and are shown for each model as well as AIC_c_ and ΔAIC_c_ (difference in AIC_c_ from the global model). ‘Precipitation’ is the average daily precipitation per site from October 1 to September 30 and ‘Temperature’ is the average daily maximum temperature for this same time period. ‘Impervious surface’ was measure in a 2 km radius around each site and year.

### Community composition and biotic homogenization

**Fig 3:**
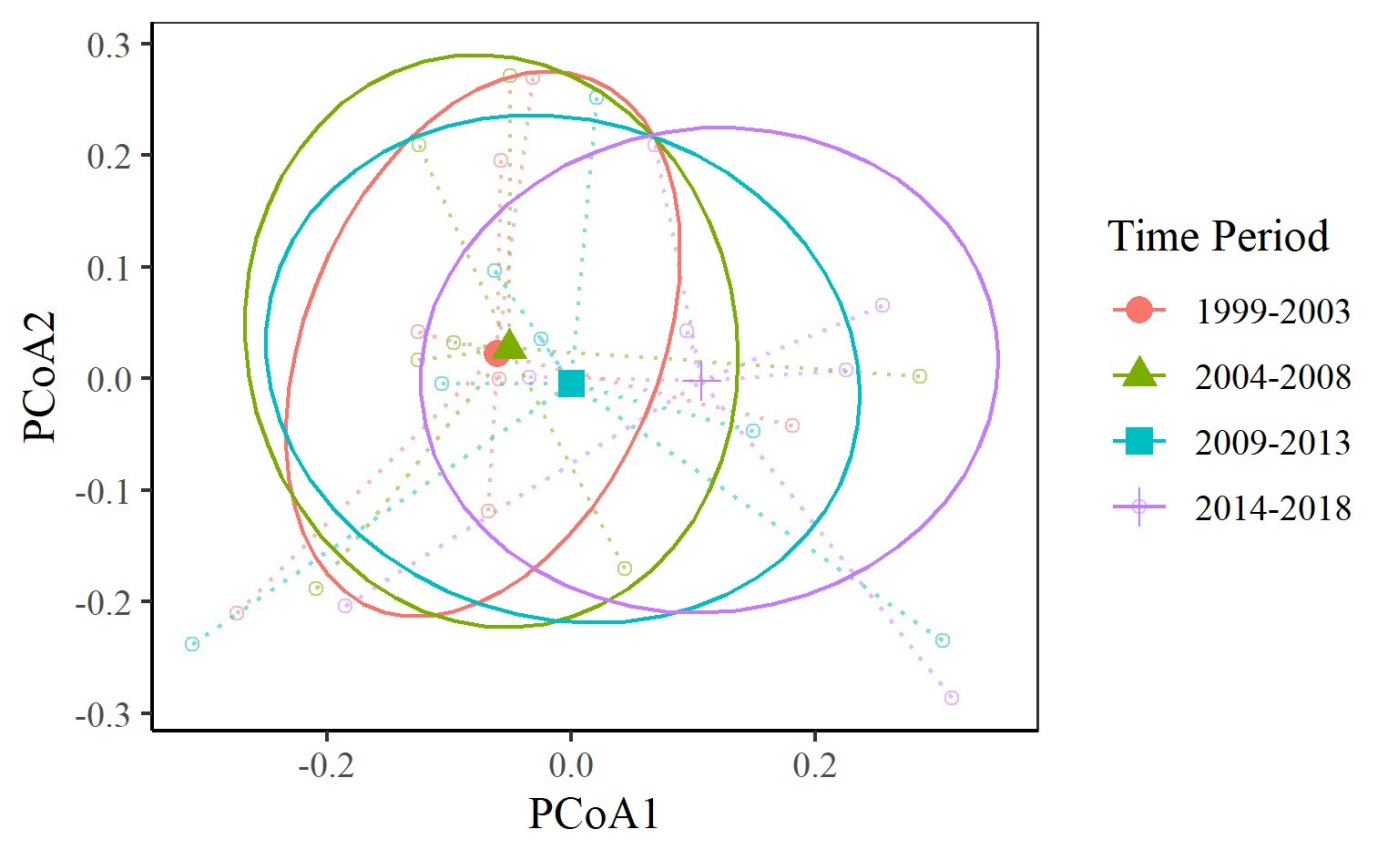
Ordination of butterfly community composition change through time. Larger filled symbols are the group centroid for all sites in each time period (1999–2003, 2004–2008, 2009–2013, 2014–2018). Empty dots are site centroids for each time period. Ellipses represent one standard deviation from the group centroids. The colors correspond to the time period. Our analyses show that the group centroids have shifted (i.e. the communities have changed), but that the dispersion of sites around the centroids (i.e. homogenization) has not changed between time periods.

The PERMANOVA model explained 42% of the variation in the butterfly community and revealed that butterfly community composition changed over time (Table 4). Differences in temperature, impervious surface cover, and time period explained 16.6%, 14.6%, and 9.7% of the variation in butterfly communities, respectively. Although temperature explained most of the variation in the butterfly community, it did not differentiate communities between the four time periods. Precipitation did not have a significant effect on variation in butterfly communities. Although communities shifted through time, we found no evidence of overall biological homogenization (F = 0.012, p = 0.998) (Fig 3).

**Table 4:** Results of PERMANOVA examining changes in butterfly community composition and potential drivers at seven sites in Illinois during four time periods (2001, 2006, 2011, 2016).

Source	DF	SS	MS	Pseudo F	*p*
Time Period	3	0.36	0.12	1.17	<0.01
Temperature	1	0.62	0.62	6.03	0.14
Precipitation	1	0.06	0.06	0.54	0.91
Impervious surface	1	0.55	0.55	5.29	<0.05
Residuals	21	2.17	0.10		
Total	27	3.77			

PERMANOVA R^2^ = 42.3. ‘Precipitation’ is the average daily precipitation per site from October 1 to September 30 and ‘Temperature’ is the average daily maximum temperature for this same time period.

## Discussion

Our data indicate significant declines in butterfly abundance and species richness over two decades. From 1999 to 2018, the linear models indicated that butterfly abundance decreased by more than half (52.5% in total, 3.8%/year) and species richness decreased by over a quarter (27.1% in total; 1.6%/year). These results are similar to long-term studies elsewhere in the United States [19, 21] and Europe [5, 11–15] that have found similarly substantial declines in butterfly abundance and species richness. Importantly, our results indicate that these declines are occurring even after accounting for changing climate and land use around the study sites.

In addition to declines in abundance and richness, we found that butterfly communities in Illinois changed in composition over time. Specifically, abundance and richness of all functional groups, except non-resident species, significantly decreased with time. This result corresponds to other studies that have found indiscriminate abundance declines across butterfly functional groups [15, 19] including widespread generalists [34]. While the non-resident butterflies did not show a significant decline over time, they were a relatively small group (only 17 of 74 species) with low abundances to begin with (abundances <5%; S1 Appendix). Therefore, the relative scarcity and high population variation of these migratory species may explain why a trend was not detected, similar to migratory patterns observed in Ohio [19].

Although most functional groups declined in abundance and/or richness over time, the functional groups that experienced the greatest abundance declines (as determined by the standardized coefficient estimate) were univoltine species (-0.36), larval host plant specialists (-0.24), and resident species (-0.23). Several regionally-common butterflies, including the Black Dash (*Euphyes conspicua*), Broken Dash (*Wallengrenia egeremet*), Eyed Brown (*Satyrodes eurydice*), Crossline Skipper (*Polites origenes*), and European Skipper (*Thymelicus lineola*) fit these criteria, and our data indicates that they may be declining at one or both of our sites experiencing significant species richness declines. However, it is important to note that many widespread generalist species such as the Black Swallowtail (*Papilio polyxenes)*, and the Buckeye (*Junonia coenia)* also appear to be declining in abundance within those sites. Even the common Cabbage White (*Pieris rapae*) seems to be declining within the IBMN monitoring system over these last 20 years (D. Taron, unpublished data). Future analyses of species-specific population trends may aid in prioritizing conservation of the most regionally imperiled species (e.g., the Regal Fritillary (*Speyeria idalia)*) [45].

Despite the changes in composition, we found no evidence of biotic homogenization (Fig 3). Biotic homogenization is independent of the number of species and typically occurs when invasive or generalist species increase at the expense of rarer or more specialized species [29–30]. The observed decline of butterflies across most functional groups, and concordant lack of increases in widespread species groups, likely explains why we did not see an increase in biotic homogenization despite a general decline in richness and abundance.

We found that butterfly species richness increased with higher temperatures. Furthermore, temperature explained a large portion of changes in butterfly composition through time. This is not surprising as many butterflies benefit from higher summer temperatures through a longer flight season, which can lead to larger populations [46] and expansion into new areas. Previous studies have found a positive association between maximum temperature and species richness [16], as well as abundance [26, 46], and also between increased temperatures and more rapid range shifts for Lepidoptera in northern latitudes [47]. Higher temperatures positively affected richness of non-resident species, in addition to larval host plant specialists and bi/multivoltine species. Based on an indicated northern range-shift in highly vagile and migratory butterflies [16, 18, 48], it is not surprising that we saw an increase in richness of these non-resident species in warmer (and less cold) years. However, it is important to note that despite variation in temperature, these and other species were not increasing through time, supporting the idea that additional anthropogenic factors may be negating any positive responses to warming global temperatures [11].

The amount of surrounding impervious surface is commonly associated with declines in butterflies [16, 49, 50]. Surprisingly, we found a positive effect of this variable (at the 2 km radius scale) on butterfly species richness. Impervious surface also had a positive effect on the species richness of all functional groups except host plant generalists. The amount of impervious surface ranged from 14 to 34% per site and did not change through time for most of our locations. For the two sites that did experience increases in impervious surface, the shift was from agricultural to urban development, a typical pattern for this time period for Illinois, especially the urbanized northern region of the state that includes the expansive Chicago metropolitan area [51]. The site that experienced the largest increase in surrounding impervious surface (Hickory Creek Barrens) is also the site with the largest decrease in the surrounding cropland. Relative to agricultural land use, moderate levels of urbanization may hold several benefits for butterflies and other insects moving through the landscape. For example, pollinators in urbanized landscapes benefit from floral-rich private and community gardens [52, 53] as well as reduced pesticides relative to intensive agricultural fields. Furthermore, increased proximity of dense human populations may result in more consistent land management activities. Although it was not possible for us to quantify management efforts (due to variation in records, techniques, extent, and frequency), our study locations closest to Chicago have been subject to extensive restoration and environmental stewardship activities. Overall, while all locations were surrounded by anthropogenic land use, the consistent positive association with impervious surface highlights the biodiversity potential of urban areas (at least at moderate levels, e.g., the 14–34% impervious surface range of this study) relative to agricultural landscapes.

A significant interaction between time and precipitation was noted for overall butterfly abundance as well as abundance of residents, larval host plant specialists, and univoltine species. The significant interaction suggests that precipitation in recent years is having more of a negative effect on butterfly abundance, possibly due to the magnitude and timing of precipitation in recent years. Other studies have found precipitation to have negative effects [26] especially during the spring [46] and on habitat specialists [13]. Alternately, summer precipitation was found to have a positive effect on butterfly numbers in the relatively dryer western United States [21] and a continental analysis of North America indicated that butterflies are increasing in cooler, wetter regions [20]. Butterflies are sensitive to drought conditions [54], and precipitation may thus have variable effects [16] based on location and climate. Future climate predictions for the Midwestern United States include heavier precipitation in the winter and spring along with drier summers [55] and increased drought risk, with substantial variation in ecoregions across the state [56]. Thus, it will be important to continue monitoring the nuanced effects of seasonal precipitation along with other ongoing climate changes for butterfly communities.

Our models indicate general declines in butterfly abundance and richness but the exact mechanism of these declines is unclear. This is especially vexing because the usual suspects of climate variation and land use change had neutral or positive effects in our models. One possible explanation for the declines is the so-called ‘ground-hog effect’ whereby declines appear due to butterflies shifting their activity outside of a standardized sampling window in response to climate change [57]. In our case, sampling was standardized between June 1 and July 31 across the 20 years of the study. If butterflies are progressively expanding their activity window to later or earlier than this time period, there may appear to be fewer butterflies. However, if this was the case we might expect fewer butterflies in warmer years (when butterfly activity can ‘spread out’ to months beyond June and July) whereas we found the opposite pattern.

Another possible explanation for the decline of butterfly abundance and richness is the prevalence of intensive agriculture in this region, as agriculture can have adverse effects on butterflies [31, 58]. While we did not include agricultural cover in our final models (due to its negative correlation with impervious surface), the percent crop cover in this system was either very low (<1% at three sites) or declining over the study period (S3 Appendix), indicating that increases in monoculture and agriculture-associated pesticide use *during the time period of this study* are unlikely to be the direct causes of the observed declines in butterfly abundance and richness in these sites. That said, Illinois has experienced enormous increases in agriculture (and urbanization) over the last two centuries. More than half (59%) of the state’s land area was prairie in 1820 but, by 1980 prairie made up just 1% of the state’s land area, with most having been converted to agriculture or urban land use [59]. Thus, it is possible that the declines in this study represent the tail end of longer-term decreases, as has been suggested for similar studies in Europe documenting declines after major periods of land use change [5].

Invasive species in the preserves and the surrounding landscape could also indirectly negatively impact butterfly communities by affecting host plant availability [60, 61] or predation [62, 63], or by serving as ecological traps [64, 65]. Since butterfly abundance depends heavily on the availability of host plants [66], removal of invasive plants as well as maintenance and replanting of native host plants is an important aspect of management. From reviewing the management plans of each study site and directly reaching out to land managers, we know that invasive plants are present within the sites but are actively being managed and native seeds are being planted. Some studies have considered the potential for certain management actions to have short-term detrimental effects on butterflies. For example, Swengel et al. [67] found declines in Midwestern prairie specialist butterflies to be related to increased fire regimes at protected areas. Other studies, however, have shown that prescribed burns maintain host plants, such as violets [68], and possibly benefit butterflies if strategically placed and sufficiently temporally heterogeneous to allow for recolonization of butterflies at burn sites [69, 70]. The Loess smoothed plots of annual abundance and richness show steep declines at some sites (Table 1; especially Somme Prairie Grove, Bluff Spring Fen, and Ferson Creek Fen) and it would seem that these, too, could be related to changes in burn frequency or other forms of local land management. However, additional communication with land managers at five of the seven sites (and review of published management plans at all locations) did not yield compelling evidence that management actions at the sites are connected to the butterfly declines. Most sites burn relatively small and rotating areas of prairie on a 1.5 to 3-year frequency with no large-scale burns directly coinciding with declines. Furthermore, invasive species removal preceded most declines (by several years) and, in all cases, has been complemented by substantial additions of native wildflowers. In general, local management seems well-suited to support butterflies and other native pollinators.

Preserve size and habitat heterogeneity also likely play an important role in the observed trends. The two largest sites (316 and 624 ha, respectively) are the only sites that did not experience a significant decline in either species richness or abundance, and the smallest site (17 ha) was the only site to experience a reduction in both abundance and richness. Smaller habitat areas can be more affected by both vegetative disturbances and weather changes, both of which can have negative effects on butterflies [53, 71]. Preserve size is often related to habitat heterogeneity as well; heterogeneous landscapes with multiple habitat types were associated with more stable population dynamics for 35 British butterfly species [72]. Three of the four sites that experienced a decrease in butterfly abundance seem to be primarily open habitat types, including wetlands and prairies. The remaining three sites, two of which were the largest preserves, included woodlands or forests in addition to more open ecosystems. The greater variety of resources and micro-climates in large, heterogeneous preserves may help buffer populations against variation in climate and other disturbances [72, 73]. Wagner et al. [74] found that, despite general declines in moths in Europe, some local areas within individual countries still retain high levels of diversity, suggesting that regional stressors do not always operate with uniform intensity. The fact that butterflies did not decline at all seven of our study locations suggests that local factors (e.g., preserve size, habitat heterogeneity) may be capable of counteracting regional stressors such as climate and landscape change.

We restricted our study to seven consistently monitored sites so that we could evaluate changes in composition and biotic homogenization through time, in addition to evaluating trends in abundance and richness. Although the Illinois Butterfly Monitoring Network includes over 200 locations, most locations have been inconsistently monitored through time or experienced substantial local changes in transect routes, management, or observers. While rigorous methods exist to handle missing data (e.g., rTrim), there are substantial challenges to analyzing change through time (particularly for composition and biotic homogenization) given the highly heterogeneous nature of the data. For example, monitoring in early years was restricted to preserves, while the recently added sites tend to be more urban which could bias trends. Despite these challenges, it would be interesting to see if the general abundance and richness trends identified in this paper also are apparent in the wider network of monitoring locations (both in Illinois and the Midwest generally) after carefully correcting for changes in monitoring locations and variable sampling effort. Such an analysis might be able to identify critical thresholds for habitat heterogeneity, preserve area, surrounding landscape configuration/scale, and other factors related to butterfly persistence.

## Conclusions

A cumulative estimated decline of 52.5% in abundance and 27.1% in species richness from 1999 to 2018 shows that butterflies in Illinois are facing steep declines, even in managed protected areas. This is perhaps not surprising given that these locations are embedded in a highly altered urban and agricultural landscape matrix. Notably, however, our results indicate that warmer temperatures, changing precipitation, and impervious surface were probably not the causes of this decline. In fact, our data suggest that some degree of urbanization may benefit butterflies, at least relative to surrounding croplands. Furthermore, we found that butterfly declines were apparent despite the benefit of warmer years. Our study is not unique in finding evidence of declines without a clear and obvious causal mechanism [6] and this may be because multiple stressors are acting synergistically (e.g., ‘death by a thousand cuts’) [7] making it hard to isolate a single factor.

Overall, our results and those of other studies suggest that increasing the size and heterogeneity of conservation areas may be necessary to maintain butterfly communities into the future. Furthermore, site-specific management strategies may play a large role in the stability of butterfly populations. Finally, it is important to note that these and other similar study locations are not insular, but rather part of a predominantly anthropogenic landscape matrix that could be adapted to facilitate movement of butterflies among habitat patches. Further research disentangling the driving forces behind butterfly and insect declines is certainly warranted. The increasing prevalence of standardized long-term citizen science data will likely aid in this effort.

## Supporting information

S1 Appendix Abundance rank and functional group placements of each species. Residency, larval host plant specialism, and voltinism were determined using Lotts & Naberhaus 2021 (and references therein), Jeffords et al. 2019, and Bouseman et al. 2006.
(PDF)

S2 Appendix Scatterplots of butterfly richness and abundance through time (1999–2018) at each of the seven study locations. The 20-year richness and abundance trend lines were generated using loess modeling (75% of points fit, Epanechnikov kernel) (SPSS 2017, Version 25) of annual butterfly richness and abundance. The Spearman’s correlation is included in each panel (* = p<0.05. ** = p<0.01).
(PDF)

S3 Appendix Changes in percent impervious and percent crop. Percent impervious and percent crop (2 km radius) for seven study sites in Illinois from 2001 to 2016. ‘% Change’ indicates the change from 2001 to 2016.
(PDF)

S4 Appendix All initial variable pairs ranked by abstolute repeated measures correlation value. Average temperature minimum (Avg temp min) and crop cover were removed from analyses due to their respectively high correlations with average temperature maximum (Avg temp max) and impervious surface.
(PDF)

S1 Data (XLSX)

S1 File (ZIP)
