# Supplementary material for: Butterfly declines in protected areas of Illinois: Assessing the influence of two decades of climate and landscape change
Source: PLoS One. 2021 Oct 13;16(10):e0257889. doi: 10.1371/journal.pone.0257889 (PMC8513915; doi:10.1371/journal.pone.0257889)
Supplement: S1 Appendix — Residency, larval host plant specialism, and voltinism were determined using Lotts & Naberhaus 2021 (and references therein), Jeffords et al. 2019, and Bouseman et al. 2006. (PDF) [file pone.0257889.s001.pdf]

S1 Appendix: Abundance rank and functional group placements of each species. Residency, larval host plant specialism, and voltinism were determined using Lotts & Naberhaus 2021 (and references therein), Jeffords et al. 2019, and Bouseman et al. 2006.

Rank	Species Name	Scientific Name	Total Abundance	Percent	Resident or Non-resident	Host plant Specificity	Univoltine or Multivoltine?
1	Cabbage White	*Pieris rapae*	7254	23	Resident	Generalist	Multivoltine
2	Common Wood-Nymph	*Cercyonis pegala*	2988	9	Resident	Specialist	Univoltine [a]
3	Pearl Crescent	*Phyciodes tharos*	2698	9	Resident	Specialist	Multivoltine
4	Great Spangled Fritillary	*Speyeria cybele*	2122	7	Resident	Specialist	Univoltine
5	Spring/Summer Azure	*Celastrina ladon/neglecta*	2102	7	Resident	Generalist	Multivoltine [b]
6	Clouded/Orange Sulphur	*Colias philodice/eurytheme*	2023	6	Resident	Specialist	Multivoltine
7	Eastern Tailed-Blue	*Everes comyntas*	1581	5	Resident	Specialist	Multivoltine
8	Little Wood-Satyr	*Megisto cymela*	1445	5	Resident	Specialist	Univoltine
9	Monarch	*Danaus plexippus*	1213	4	Non-resident	Specialist	Multivoltine
10	Eyed Brown	*Satyrodes eurydice*	1038	3	Resident	Specialist	Univoltine
11	Red Admiral	*Vanessa atalanta*	724	2	Non-resident	Specialist	Multivoltine
12	Northern Pearly-Eye	*Enodia anthedon*	658	2	Resident	Specialist	Multivoltine
13	Black Swallowtail	*Papilio polyxenes*	496	2	Resident	Generalist	Multivoltine
14	Baltimore Checkerspot	*Euphydryas phaeton*	439	1	Resident	Generalist	Univoltine
15	Eastern Tiger Swallowtail	*Papilio glaucus*	439	1	Resident	Generalist	Multivoltine
16	Wild Indigo Duskywing	*Erynnis baptisiae*	430	1	Resident	Specialist	Multivoltine
17	Black Dash	*Euphyes conspicua*	366	1	Resident	Specialist	Univoltine
18	Viceroy	*Limenitis archippus*	318	1	Resident	Specialist	Multivoltine
19	Least Skipper	*Ancyloxypha numitor*	291	1	Resident	Specialist	Multivoltine
20	Red-spotted Purple	*Limenitis arthemis astyanax*	241	0.8	Resident	Generalist	Multivoltine
21	American Lady	*Vanessa virginiensis*	224	0.7	Non-resident	Specialist	Multivoltine
22	Appalachian Brown	*Satyrodes appalachia*	222	0.7	Resident	Specialist	Univoltine [c]
23	Peck's Skipper	*Polites peckius*	196	0.6	Resident	Specialist	Multivoltine
24	Silvery Checkerspot	*Chlosyne nycteis*	193	0.6	Resident	Generalist	Multivoltine
25	Question Mark	*Polygonia interrogationis*	191	0.6	Resident	Generalist	Multivoltine

26	Silver-spotted Skipper	*Epargyreus clarus*	185	0.6	Resident	Specialist	Multivoltine
27	Banded Hairstreak	*Satyrium calanus*	169	0.5	Resident	Specialist	Univoltine
28	Buckeye	*Junonia coenia*	149	0.5	Non-resident	Generalist	Multivoltine
29	Eastern Comma	*Polygonia comma*	145	0.5	Resident	Generalist	Multivoltine
30	Mourning Cloak	*Nymphalis antiopa*	121	0.4	Resident	Generalist	Univoltine
31	European Skipper	*Thymelicus lineola*	100	0.3	Resident	Specialist	Univoltine
32	Dun Skipper	*Euphyes vestris*	99	0.3	Resident	Specialist	Multivoltine [d]
33	Hobomok Skipper	*Poanes hobomok*	87	0.3	Resident	Specialist	Univoltine
34	Delaware Skipper	*Anatrytone logan*	77	0.2	Resident	Specialist	Multivoltine
35	Tawny-edged Skipper	*Polites themistocles*	67	0.2	Resident	Specialist	Multivoltine
36	Northern Broken-Dash	*Wallengrenia egeremet*	53	0.2	Resident	Specialist	Univoltine
37	Coral Hairstreak	*Satyrium titus*	47	0.1	Resident	Specialist	Univoltine
38	Little Yellow	*Eurema lisa*	44	0.1	Non-resident	Specialist	Multivoltine
39	Mulberry Wing	*Poanes massasoit*	38	0.1	Resident	Specialist	Univoltine
40	Southern Dogface	*Colias cesonia*	33	0.1	Non-resident	Specialist	Univoltine
40	Little Glassywing	*Pompeius verna*	33	0.1	Resident	Specialist	Univoltine
42	Giant Swallowtail	*Papilio cresphontes*	32	0.1	Resident	Specialist	Multivoltine
43	Fiery Skipper	*Hylephila phyleus*	30	0.1	Non-resident	Specialist	Multivoltine
44	American Snout	*Libytheana carinenta*	25	0.1	Non-resident	Specialist	Multivoltine
45	Hackberry Emperor	*Asterocampa celtis*	23	0.1	Resident	Specialist	Multivoltine
46	Common Roadside-Skipper	*Amblyscirtes vialis*	21	0.1	Resident	Specialist	Univoltine
47	Painted Lady	*Vanessa cardui*	18	0.05	Non-resident	Generalist	Multivoltine
48	Common Sootywing	*Pholisora catullus*	17	0.05	Non-resident	Specialist	Multivoltine
49	Dion Skipper	*Euphyes dion*	11	>0.05	Resident	Specialist	Univoltine
49	Aphrodite Fritillary	*Speyeria aphrodite*	11	>0.05	Resident	Specialist	Univoltine
51	Crossline Skipper	*Polites origenes*	10	>0.05	Resident	Specialist	Multivoltine
51	Variegated Fritillary	*Euptoieta claudia*	10	>0.05	Non-resident	Generalist	Multivoltine
53	Acadian Hairstreak	*Satyrium acadica*	9	>0.06	Resident	Specialist	Univoltine
53	Bronze Copper	*Lycaena hyllus*	9	>0.07	Resident	Specialist	Multivoltine
53	Striped Hairstreak	*Satyrium liparops*	9	>0.08	Resident	Generalist	Univoltine
56	Cloudless Sulphur	*Phoebis sennae*	7	>0.025	Non-resident	Specialist	Multivoltine

57	Long Dash	*Polites mystic*	6	>0.025	Resident	Specialist	Univoltine
58	Funereal Duskywing	*Erynnis funeralis*	4	>0.026	Non-resident	Specialist	Multivoltine
58	Gray Comma	*Polygonia progne*	4	>0.027	Resident	Generalist	Multivoltine
60	Broadwinged Skipper	*Poanes viator*	3	>0.01	Resident	Specialist	Univoltine
60	Dainty Sulphur	*Nathalis iole*	3	>0.01	Non-resident	Specialist	Multivoltine
60	Gorgone Checkerspot	*Chlosyne gorgone*	3	>0.01	Resident	Specialist	Multivoltine
60	Gray Hairstreak	*Strymon melinus*	3	>0.01	Resident	Generalist	Multivoltine
60	Zabulon Skipper	*Poanes zabulon*	3	>0.01	Resident	Specialist	Multivoltine
65	Clouded Skipper	*Lerema accius*	2	>0.01	Non-resident	Specialist	Multivoltine
65	Southern Cloudywing	*Thorybes bathyllus*	2	>0.01	Resident	Specialist	Univoltine
67	Common Checkered-Skipper	*Pyrgus communis*	1	>0.01	Non-resident	Specialist	Multivoltine
68	Checkered White	*Pontia protodice*	1	>0.01	Non-resident	Generalist	Multivoltine
69	Harvester	Feniseca tarquinius	1	>0.01	Resident	Generalist	Multivoltine
70	'Northern' Oak Hairstreak	*Satyrium favonius ontario*	1	>0.01	Resident	Specialist	Univoltine
71	Pipevine Swallowtail	*Battus philenor*	1	>0.01	Resident	Specialist	Multivoltine
72	Swamp Metalmark	*Calephelis muticua*	1	>0.01	Resident	Specialist	Univoltine [e]
73	Tawny Emperor	*Asterocampa clyton*	1	>0.01	Resident	Specialist	Univoltine
74	White-M Hairstreak	*Parrhasius m album*	1	>0.01	Resident	Specialist	Multivoltine

[a]Jeffords et al. 2019 describe *Cercyonis pegala (*Common Wood-nymph) as having one long emergence. We classified this as univoltine.

[b]*Celastrina ladon (Spring Azure)* is univoltine while *C. neglecta* (Summer Azure) is multivoltine. Because the vast majority of observations in this dataset are likely of *C. neglecta* (D. Taron, personal observation), we classified this taxa as multivoltine.

[c] There are two subspecies of *Satyrodes appalachia* (Appalachian Brown) in Illinois. The subspecies found at all of the sites in the analysis is univoltine so we used this classification for voltinism. The other subspecies is in the far southern tip of the state.

[d] Personal observations (D. Taron) *Euphyes vestris (*Dun Skipper) led us to classify this species as multivoltine for this region.

[e]Jeffords et al. 2019 states that two generations occur in central Illinois but one in northern Illinois where this study occurred.
