# Supplementary material for: Butterfly declines in protected areas of Illinois: Assessing the influence of two decades of climate and landscape change
Source: PLoS One. 2021 Oct 13;16(10):e0257889. doi: 10.1371/journal.pone.0257889 (PMC8513915; doi:10.1371/journal.pone.0257889)
Supplement: S2 Appendix — The 20-year richness and abundance trend lines were generated using loess modeling (75% of points fit, Epanechnikov kernel) (SPSS 2017, Version 25) of annual butterfly richness and abundance. The Spearman’s correlation is included in each panel (* = p<0.05. ** = p<0.01). (PDF) [file pone.0257889.s002.pdf]

S2 Appendix: Scatterplots of butterfly richness and abundance through time (1999-2018) at each of the seven study locations. The 20-year richness and abundance trend lines were generated using loess modeling (75% of points fit, Epanechnikov kernel) (SPSS 2017, Version 25) of annual butterfly richness and abundance. The Spearman's correlation is included in each panel (* = $p<0.05$. ** = $p<0.01$).

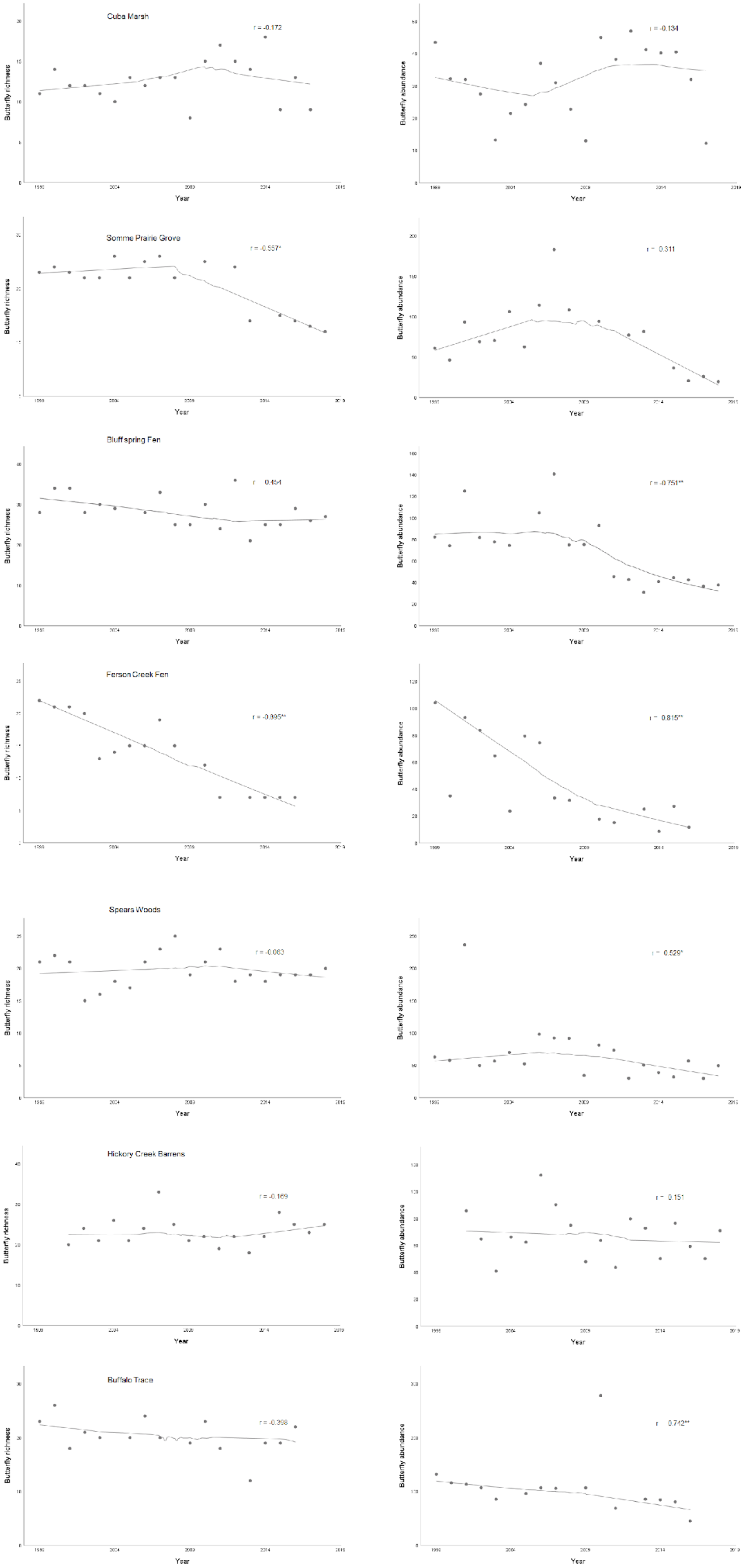
Cuba Marsh
r = -0.172
Butterfly richness
Year
r = -0.134
Butterfly abundance
Year
Somme Prairie Grove
r = -0.557*
Butterfly richness
Year
0.311
Butterfly abundance
Year
Bluff spring Fen
0.454
Butterfly richness
Year
r = -0.751**
Butterfly abundance
Year
Ferson Creek Fen
r = -0.895**
Butterfly richness
Year
0.815**
Butterfly abundance
Year
Spears Woods
r = -0.063
Butterfly richness
Year
0.529*
Butterfly abundance
Year
Hickory Creek Barrens
r = -0.169
Butterfly richness
Year
0.151
Butterfly abundance
Year
Buffalo Trace
r = -0.398
Butterfly richness
Year
0.742**
Butterfly abundance
Year
