# Supplementary material for: Butterfly declines in protected areas of Illinois: Assessing the influence of two decades of climate and landscape change
Source: PLoS One. 2021 Oct 13;16(10):e0257889. doi: 10.1371/journal.pone.0257889 (PMC8513915; doi:10.1371/journal.pone.0257889)
Supplement: S3 Appendix — Percent impervious and percent crop (2 km radius) for seven study sites in Illinois from 2001 to 2016. ‘% Change’ indicates the change from 2001 to 2016. (PDF) [file pone.0257889.s003.pdf]

S3 Appendix: Percent impervious and percent crop (2 km radius) for seven study sites in Illinois from 2001 to 2016. '% Change' indicates the change from 2001 to 2016.

Site	*2001*	*2006*	*2011*	*2016*	*% Change*
Bluff Spring Fen					
% Impervious	25.8	31.9	32.6	34.1	+8.3
% Crop	5.0	3.2	3.1	2.7	-2.3
Buffalo Trace					
% Impervious	14.3	14.9	15.2	15.7	+1.4
% Crop	25.9	23.6	23.3	22.3	-3.6
Cuba Marsh					
% Impervious	19.9	20.3	20.5	20.6	+0.7
% Crop	0.1	0.2	0.2	0.3	+0.2
Ferson Creek Fen					
% Impervious	17.2	17.3	17.5	17.6	+0.4
% Crop	1.5	1.4	1.4	0.4	-1.1
Hickory Creek Barrens					
% Impervious	17.3	25.7	26.5	26.7	+9.3
% Crop	17.1	6.3	5.7	5.8	-11.3
Somme					
% Impervious	32.9	33.4	33.4	33.5	+0.6
% Crop	0.3	0.3	0.3	0.3	0
Spears Woods					
% Impervious	21.1	21.2	21.4	21.5	+0.4
% Crop	0.1	0.1	0.1	0.1	0
