# Supplementary material for: Butterfly declines in protected areas of Illinois: Assessing the influence of two decades of climate and landscape change
Source: PLoS One. 2021 Oct 13;16(10):e0257889. doi: 10.1371/journal.pone.0257889 (PMC8513915; doi:10.1371/journal.pone.0257889)
Supplement: S4 Appendix — Average temperature minimum (Avg temp min) and crop cover were removed from analyses due to their respectively high correlations with average temperature maximum (Avg temp max) and impervious surface. (PDF) [file pone.0257889.s004.pdf]

S4 Appendix. All initial variable pairs ranked by abstolute repeated measures correlation value. Average temperature minimum (Avg temp min) and crop cover were removed from analyses due to their respectively high correlations with average temperature maximum (Avg temp max) and impervious surface. Asterisks denote significance as follows: * = <0.05, ** = <0.01, *** = <0.001.

Variable Pair	Correlation
Avg temp max <-> Avg temp min	0.923***
Impervious surface <-> Crop cover	-0.831***
Impervious surface <-> Time	0.527***
Abundance <-> Richness	0.494***
Crop cover <-> Time	-0.409***
Richness <-> Time	-0.403***
Avg precipitation <-> Time	0.399***
Abundance <-> Time	-0.389***
Richness <-> Avg temp max	0.274**
Impervious surface <-> Avg precipitation	0.252**
Avg precipitation <-> Avg temp max	-0.225*
Crop cover <-> Avg precipitation	-0.221*
Avg temp max <-> Time	-0.174
Richness <-> Avg temp min	0.154
Abundance <-> Impervious surface	-0.139
Richness <-> Avg precipitation	-0.133
Richness <-> Impervious surface	-0.109
Impervious surface <-> Avg temp max	-0.077
Abundance <-> Avg precipitation	-0.066
Abundance <-> Crop cover	0.059
Avg precipitation <-> Avg temp min	-0.058
Impervious surface <-> Avg temp min	0.057
Crop cover <-> Avg temp min	-0.056
Avg temp min <-> Time	0.051
Crop cover <-> Avg temp max	0.045
Abundance <-> Avg temp max	0.043
Richness <-> Crop cover	0.033
Abundance <-> Avg temp min	0.022
